# The Effects of FreeSurfer Version, Workstation Type, and Macintosh Operating System Version on Anatomical Volume and Cortical Thickness Measurements

**DOI:** 10.1371/journal.pone.0038234

**Published:** 2012-06-01

**Authors:** Ed H. B. M. Gronenschild, Petra Habets, Heidi I. L. Jacobs, Ron Mengelers, Nico Rozendaal, Jim van Os, Machteld Marcelis

**Affiliations:** 1 Department of Psychiatry and Neuropsychology, School for Mental Health and Neuroscience, Maastricht University Medical Center, Maastricht, Alzheimer Center Limburg, The Netherlands; 2 European Graduate School of Neuroscience (EURON), Maastricht University, Maastricht, The Netherlands; 3 Cognitive Neurology Section, Institute of Neuroscience and Medicine-3, Research Centre Jülich, Jülich, Germany; 4 King's College London, King's Health Partners, Department of Psychosis Studies Institute of Psychiatry, London, United Kingdom; Wake Forest School of Medicine, United States of America

## Abstract

FreeSurfer is a popular software package to measure cortical thickness and volume of neuroanatomical structures. However, little if any is known about measurement reliability across various data processing conditions. Using a set of 30 anatomical T1-weighted 3T MRI scans, we investigated the effects of data processing variables such as FreeSurfer version (v4.3.1, v4.5.0, and v5.0.0), workstation (Macintosh and Hewlett-Packard), and Macintosh operating system version (OSX 10.5 and OSX 10.6). Significant differences were revealed between FreeSurfer version v5.0.0 and the two earlier versions. These differences were on average 8.8±6.6% (range 1.3–64.0%) (volume) and 2.8±1.3% (1.1–7.7%) (cortical thickness). About a factor two smaller differences were detected between Macintosh and Hewlett-Packard workstations and between OSX 10.5 and OSX 10.6. The observed differences are similar in magnitude as effect sizes reported in accuracy evaluations and neurodegenerative studies.

The main conclusion is that in the context of an ongoing study, users are discouraged to update to a new major release of either FreeSurfer or operating system or to switch to a different type of workstation without repeating the analysis; results thus give a quantitative support to successive recommendations stated by FreeSurfer developers over the years. Moreover, in view of the large and significant cross-version differences, it is concluded that formal assessment of the accuracy of FreeSurfer is desirable.

## Introduction

FreeSurfer (Athinoula A. Martinos Center for Biomedical Imaging, Harvard-MIT, Boston) comprises a popular and freely available set of tools for deriving neuroanatomical volume and cortical thickness measurements from automated brain segmentation (http://surfer.nmr.mgh.harvard.edu), recently summarised by Fischl [1]. A number of reported studies discussed the accuracy of the technique by comparing the volume of specific brain structures, such as the hippocampus or amygdala, with manually derived volumes [2]–[5]. The measurement of cortical thickness was validated against histological analysis [6] and manual measurements [7], [8]. Also the reliability of the measurements was subject of a number of investigations. Some of these studies addressed the effect of scanner-specific parameters, including field strength, pulse sequence, scanner upgrade, and vendor (cortical thickness: [9], [10]; volume: [11]). In addition, the scan-rescan variability of a number of subcortical brain volumes was assessed [12]–[14]. Finally, it has been shown that Freesurfer is capable of reliably capturing (subtle) morphological and pathological changes in the brain (e.g., [5], [13]).

Since FreeSurfer is CPU-intensive (20–30 hours per brain for a full segmentation is not exceptional), it is common practice to distribute the computational load among the available central processor units (CPUs) on a single workstation and/or among several workstations. Given this context, a number of questions suggest themselves: (1) does every CPU produce the same results; (2) is there any interaction between the processes running simultaneously on the same workstation; (3) does every workstation produce the same results?

Just like similar neuroimaging packages, new releases of FreeSurfer are issued regularly, fixing known bugs and improving existing tools and/or adding new ones. Each release is accompanied with documentation describing the changes relative to the previous release (http://surfer.nmr.mgh.harvard.edu/fswiki/ReleaseNotes). However, transition to a new release during the course of a study may affect the results and is therefore discouraged by the developers of FreeSurfer. This potential source of variation in outcome may invalidate comparisons between different studies. As yet, the sources and effect sizes of these variations have never been investigated in detail.

A related question is whether differences in the results may arise due to different releases of the operating system (OS).

The goal of the present study was to address the above mentioned questions by repeating the automated segmentation on the same workstation and on different workstations using a set of 30 anatomical T1-weighted MRI scans. Three different versions of FreeSurfer were used on a single Hewlett-Packard workstation and several Macintosh workstations running under two different OSX versions. In particular, we aimed to get insight into the variabilities resulting from these different data processing conditions and to compare these with reported accuracy and reliability results and morphological and pathological cerebral changes. Although the developers of FreeSurfer have been explicitly recommending users not to mix FreeSurfer versions, platforms, and OS versions within a study (see public archives at http:/www.mail-archive.com/freesurfernmr.mgh.harvard.edu), to our knowledge this is the first time that the effects of these different processing conditions have been quantified systematically.

## Materials and Methods

### Ethics statement

The study was approved by the ethics committee of the Maastricht University Medical Center and all participants gave written informed consent in accordance with the committee's guidelines and with the Declaration of Helsinki [15]. All patients were mentally competent to consent as evaluated by trained psychology graduates during the screening and informed consent procedures, i.e., participating patients were fully understanding information disclosures and study procedures.

### MRI acquisition

MRI scans were acquired with a 3.0 T Siemens Allegra MRI scanner (Siemens Medical Systems, Erlangen, Germany). Coronal T1-weighted images were obtained using an ADNI MP-RAGE sequence with TR = 2250 msec, TE = 2.6 msec, and a flip angle of 9°. The number of slices was 192 and slice thickness 1.0 mm with no interslice gap. The image matrix was 256×256 and the field of view 256×256 mm. The resulting voxel size was 1.0×1.0×1.0 mm^3^.

### Participants

For the current study, data from an ongoing longitudinal MRI study were used [16]. From a large sample consisting of 89 patients with psychotic disorder, 98 siblings of patients with psychotic disorder, and 87 controls, a total of 30 participants were randomly drawn, 10 out of each group. The age (years) of the individuals was 28.1±5.3 (range 23–38), 29.4±9.8 (range 17–43), and 28.6±11.6 (range 19–50), respectively.

### Workstations

Two workstations and corresponding operating systems were at our disposal for this study (Table 1). On the Macintosh (Mac) platforms, FreeSurfer used the UNIX shell while on the Hewlett-Packard (HP) platform, LINUX was used (CentOS 5.3). One Mac workstation was configured to run under two different OS versions by means of an external disk. Although OSX 10.6 is able to run in 64 bits mode, we used 32 bits mode only (see next section). By contrast, on the HP platform, CentOS was used in 64 bits mode.

**Table 1:** Workstations used in this study.

Name	Type	OS	CPU	N^a^	RAM
iMac1	iMac	OS X 10.5.8	3.06 GHz Intel Core Duo	2	8 GB 1067 MHz DDR3
iMac2	iMac	OS X 10.6.5	2.8 GHz Core i7	8	16 GB 1067 MHz DDR3
MacPro1	MacPro	OS X 10.5.8/10.6.5^b^	2×3.2 GHz Quad-Core Intel Xeon	8	16 GB 800 MHz DDR2
MacPro2	MacPro	OS X 10.6.4	2×3.0 GHz Quad-Core Intel Xeon	8	16 GB 1066 MHz DDR3
MacPro3	MacPro	OS X 10.6.4	2×2.6 GHz Quad-Core Intel Xeon	8	16 GB 1066 MHz DDR3
HP	HP	CentOS 5.3	2×2.66 GHz Quad-Core Intel Xeon	8	16 GB 667 MHz DDR2

a N is the number of processors.

b By means of an external disk this workstation could run under two different OS versions.

Note: All Macintosh workstations used the UNIX shell and the Hewlett-Packard (HP) workstation the LINUX shell. OSX 10.6.4/10.6.5 was used in 32 bits mode, whereas CentOS was used in 64 bits mode.

### FreeSurfer

The FreeSurfer analysis pipeline comprises two main processing streams, a volume-based stream and a surface-based stream. The volume-based stream is designed to assign a neuroanatomical label to each (sub)cortical voxel, whereas the surface-based stream is developed to derive the white and pial surfaces from which, among others, cortical volumes and cortical thickness (CT) are derived. More details can be found in Document S1 and references [2], [17]–[28].

The volumes are presented by FreeSurfer in the form of tables and labeled voxels. The tabulated volumes are more accurate than the voxel volumes because they are corrected for partial volume effects. Both types of volumes were used in our analysis (see Document S1 for more details).

In order to compare the results with accuracy results previously reported by Lehmann and colleagues [5], a few white matter and grey matter regions were merged to produce a whole gyrus or lobe (left and right), such as medial-inferior temporal gyrus (MITG), superior temporal gyrus (STG), and temporal lobe (TempL). Simarly, total ventricle volume (Ventr) was constructed (left+right added together). In this manner, a total of 7 composite volumes were assembled. (The respective segmentation labels were kindly provided to us by Dr. Manja Lehmann, University College London, UK, see Document S1 for more details).

In total, we computed 190 (sub)cortical volumes (185 for v5.0.0) and 68 CT values. It should be noted that no manual corrections were made to any of the FreeSurfer results in order to ensure a valid analysis. However, a visual inspection was performed to check the segmentations.

Three versions of FreeSurfer were used: v4.3.1, released on 19 May 2009, version v4.5.0, released on 11 August 2009, and version v5.0.0, released on 16 August 2010. For the Mac workstations these are 32 bits versions (due to problems to build some third party libraries in 64 bits mode on the Mac), whereas for the HP workstation these are 64 bits versions.

### The experiments

A number of experiments were carried out to examine the variability of the results due to different data processing conditions by repeating the data processing (“run”) on the same dataset:

1. Difference between repeated single runs on Mac and HP workstations
2. Effect of parallel runs using eight processors on Mac and HP workstations
3. Difference between runs with v4.3.1, v4.5.0, and v5.0.0 on Mac and HP workstations
4. Difference between runs with OSX 10.6.4/5 and OSX 10.5.8 on Mac workstations

Each run was started by opening a terminal window which initialised the environmental variables, followed by issuing the FreeSurfer “recon-all” command to start the data processing stream. A single run meant that only one stream was active at any time. Parallel runs on eight processors involved opening of eight terminal windows and issuing in each window the recon-all command. This resulted in eight instances of the FreeSurfer pipeline being active at the same time during which all available CPU power was mobilised. Obviously, on the iMac with two processors only two streams could be active at the same time.

The experiments where carried out on multiple Mac workstations and a single HP workstation. Experiments 1 and 2 were designed (1) to disclose any difference between runs on the same workstation and between runs on different workstations and (2) to reveal any interference between parallel running streams. Experiment 3 provided insight into the effects of different versions of FreeSurfer. In experiments 1 to 3, OSX 10.5.8 was used on the Mac workstations. Finally, the effects of different Mac OSX versions were investigated in experiment 4 for all FreeSurfer versions used.

### Statistical measures

Several statistical measures were employed to quantify the effects studied, such as mean difference and standard deviation, and the coefficient of variation (COV), defined as the percentage standard deviation relative to the mean. In addition, we computed the measure of spatial overlap of a structure, also known as similarity index (SI) or Dice coefficient [29]. Its range is between 0 (no overlap) and 1 (complete overlap). Finally, the intra-class correlation coefficient, ICC, based on the one-way random effects model [30] was calculated. More details can be found in Document S1.

For the statistical analysis, the paired Student *t* test was applied, since each time the outcome of two conditions was compared. We considered two levels of statistical significance, both corrected for multiple comparisons. The first level was set to *p*<0.05/*N*, where *N* is the number of tested volume or CT measurements (classical Bonferroni correction). For the second level, the False Discovery Rate (FDR) method [31] was applied, which is less stringent than the classical Bonferroni correction. With regard to cortical thickness, *N* equaled 68. With respect to volumes, *N* depended on the type of volume (tabulated or voxel) and the considered comparison, where we excluded the 7 composite volumes because of their dependence on the other volumes. For versions v4.3.1 and 4.5.0, *N* was 183/182 (tabulated/voxel) and for version v5.0.0, *N* was 178/178. In case of a comparison of version v4.3.1 or v4.5.0 with v5.0.0 a total of 176/178 common volumes existed.

## Results

No differences were detected between repeated single runs nor between single runs and parallel runs on the same workstation and for the same FreeSurfer and OS version. For the same OS version, all Mac workstations produced identical results. However, differences were revealed between:

Mac and HP workstations
FreeSurfer versions v4.3.1, v4.5.0, and v5.0.0
OSX 10.5.8 and OSX 10.6.4/5

Since we did not find any differences between OSX 10.6.4 and OSX 10.6.5, we will use the terms OSX 10.5 and OSX 10.6 henceforth for OSX 10.5.8 and OSX 10.6.4/5, respectively.

The differences are presented in more detail below, starting with an overview and subsequently zooming in on specific structures and data processing comparisons. For the volume measurements, only voxel volumes are considered since the results for tabulated volumes were very similar.

### Significance of result differences

**Figure 1:**
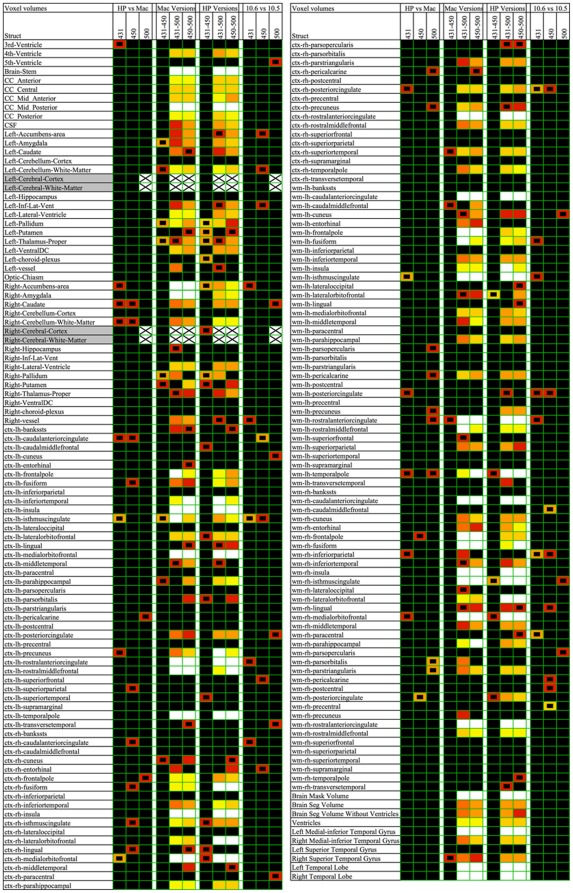
Overview of the statistical significance of voxel volume comparisons for all considered structures. Each cell is color-coded according to the value of −log10(*p*), ranging from black (*p*>0.05) to white (*p*≤0.00001), see Figure 2 for the color coding scale. The first three columns show the results obtained by comparing HP with Mac workstation for FreeSurfer versions v4.31, v4.5.0, and v5.0.0, respectively. The *p* values for the differences between the versions v4.3.1, v4.5.0 and v5.0.0 are shown in columns 4 to 6 for the Mac and in columns 7 to 9 for the HP, respectively. Finally, the last three columns refer to the contrast between OSX 10.6 and OSX 10.5 for the three considered FreeSurfer versions. Cells with a small black rectangle inside denote differences which are not significant anymore after FDR correction for multiple comparisons. White cells with an “X” represent structures for which no comparison could be made, such as left and right cerebral cortex and left and right cerebral white matter, because these are no longer available in FreeSurfer v5.0.0. In the heading row, the labels 431, 450, and 500 denote FreeSurfer v4.3.1, v4.5.0, and v5.0.0, respectively.

**Figure 2:**
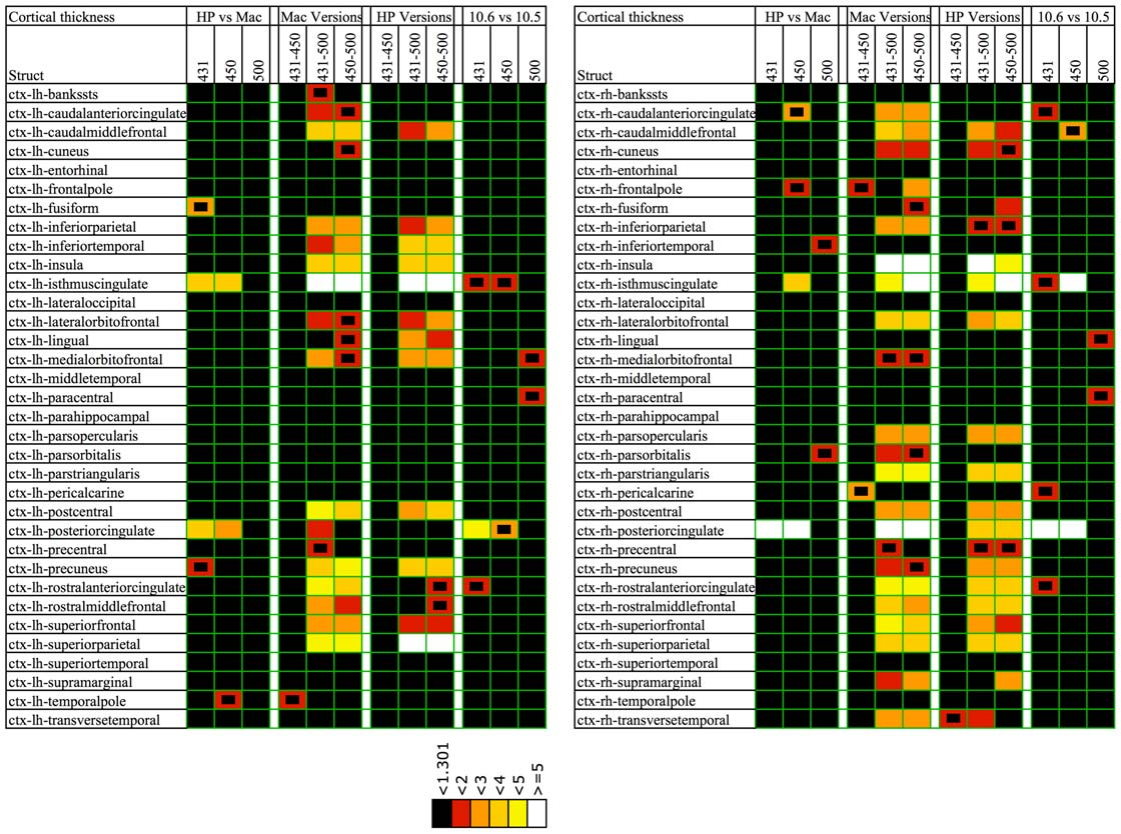
The same as **Figure 1**, but now for the cortical thickness comparisons. The color coding is represented in 6 categories of −log10(*p*), in short lnp: black for lnp<1.301 (*p*>0.05); red for lnp<2 (*p*>0.01); orange for lnp<3 (*p*>0.001); gold for lnp<4 (*p*>0.0001); yellow for lnp<5 (*p*>0.00001); white for lnp≥5 (*p*≤0.00001).

A complete overview in the form of colored cells for all comparisons is illustrated in Figure 1 (voxel volume) and Figure 2 (CT). With such reproduction, reminiscent of a DNA microarray, one can get a good impression of the results at a glance. By far the most colored cells were found for the cross-version comparisons (v4.3.1 vs. v5.0.0 and v4.5.0 vs. v5.0.0).

As described above, the level of statistical significance after correction for multiple comparisons depends on the data processing contrast being considered, see Table 2. It turned out that for the volumes, significant differences were derived only for the cross-version contrasts v5.0.0 vs. the two earlier versions (both Mac and HP). However, for CT, significant differences were found also for some other data processing comparisons. If FDR correction was applied to the volume results, then almost all the colored cells for the cross-version contrasts v5.0.0 vs. v4.3.1/v4.5.0 (both Mac and HP) in Figure 1 represent significant differences. In fact, about half of the 178 structures were significant. With a conservative Bonferroni correction, about a quarter of the structures were significant. For CT rather similar results were obtained: about half/a quarter of the 68 cortical structures were significant after FDR/Bonferroni correction in case of the cross-version contrasts v5.0.0 vs. v4.3.1/v4.5.0 (both Mac and HP). Furthermore, in case of the HP vs. Mac and Mac OSX 10.6 vs. OSX 10.5 contrasts, significant CT differences were found for versions v4.3.1 and v4.5.0, whereas no significant CT differences were present at all for version v5.0.0.

**Table 2:** Correction for multiple comparisons on volume and cortical thickness differences.

		Bonferroni correction			FDR correction		
Comparison	N^a^	p	−log10(p)	N sig^b^	p	−log10(p)	N sig^b^
Volume							
HP vs. Mac (OSX 10.5.8)							
v4.3.1	182	0.00027	3.5635	0	-	-	0
v4.5.0	182	0.00027	3.5635	0	-	-	0
v5.0.0	178	0.00028	3.5515	0	-	-	0
Mac (OSX 10.5.8)							
v4.3.1 vs. v4.5.0	182	0.00027	3.5635	0	-	-	0
v4.3.1 vs. v5.0.0	178	0.00028	3.5515	49	0.02472	1.6070	88
v4.5.0 vs. v5.0.0	178	0.00028	3.5515	46	0.02640	1.5783	94
HP							
v4.3.1 vs. v4.5.0	182	0.00027	3.5635	0	-	-	0
v4.3.1 vs. v5.0.0	178	0.00028	3.5515	46	0.02388	1.6220	85
v4.5.0 vs. v5.0.0	178	0.00028	3.5515	45	0.02556	1.5924	91
MacOSX10.6.4 vs. 10.5.8							
v4.3.1	182	0.00027	3.5635	0	-	-	0
v4.5.0	182	0.00027	3.5635	0	-	-	0
v5.0.0	178	0.00028	3.5515	0	-	-	0
Cortical thickness							
HP vs. Mac (OSX 10.5.8)							
v4.3.1	68	0.00074	3.1335	3	0.00221	2.6564	3
v4.5.0	68	0.00074	3.1335	3	0.00294	2.5315	4
v5.0.0	68	0.00074	3.1335	0	-	-	0
Mac (OSX 10.5.8)							
v4.3.1 vs. v4.5.0	68	0.00074	3.1335	0	-	-	0
v4.3.1 vs. v5.0.0	68	0.00074	3.1335	17	0.02500	1.6021	34
v4.5.0 vs. v5.0.0	68	0.00074	3.1335	15	0.02132	1.6711	29
HP							
v4.3.1 vs. v4.5.0	68	0.00074	3.1335	0	-	-	0
v4.3.1 vs. v5.0.0	68	0.00074	3.1335	12	0.01985	1.7022	27
v4.5.0 vs. v5.0.0	68	0.00074	3.1335	13	0.01985	1.7022	27
MacOSX10.6.4 vs. 10.5.8							
v4.3.1	68	0.00074	3.1335	2	0.00147	2.8325	2
v4.5.0	68	0.00074	3.1335	2	0.00147	2.8325	2
v5.0.0	68	0.00074	3.1335	0	-	-	0

a Number of tested structures.

b Number of significant structures after application of correction.

### Strength of result differences

A summary of a subset of descriptive statistics for all structures is given in Table 3 (voxel volume) and Table 4 (CT). More details can be found in the Supplementary material, e.g., Table S1 for voxel volumes, Table S2 for tabulated volumes, and Table S3 for cortical thickness. The largest differences (mean as well as range) were found for the cross-version contrasts v4.3.1/v4.5.0 vs. v5.0.0 (both Mac and HP). Generally, the mean (signed or absolute) differences and the COVs for these contrasts were about a factor of two larger than for the other contrasts. The differences manifested a large variation across the structures. The largest absolute volume difference was found in the 5th ventricle and Mac v4.5.0 vs. v5.0.0 contrast: about 64%. However, for the other contrasts some absolute volume differences were large too (about 40%). These findings translated to correspondingly low ICC values and overlap measures (SI). For CT, the largest absolute thickness difference was found in the right isthmus cingulate cortex and Mac v4.3.1 vs. v5.0.0 contrast: about 7.7%. Note that generally the ICC values for CT were larger than for the volume: they were all above 0.5547 compared to 0.0000 for volume measures. Of particular note is that the results of the HP vs. Mac contrast are almost identical to those of the OSX 10.6 vs. OSX 10.5 contrast, both for volume and CT values.

**Table 3:** Summary of voxel volume differences.

	Signed difference (%)		Coefficient of variation (%)^a^		Absolute difference (%)		ICC^b^		SI^c^	
Comparison	Mean (sd)	Range	Mean (sd)	Range	Mean (sd)	Range	Mean (sd)	Range	Mean (sd)	Range
HP vs. Mac (OSX 10.5.8)										
v4.3.1	0.13 (1.75)	−13.20–8.25	6.17 (5.60)	0.07–49.08	4.28 (3.94)	0.02–35.70	0.941 (0.069)	0.606–1.000	0.914 (0.044)	0.758–0.999
v4.5.0	0.00 (1.35)	−5.36–6.28	6.31 (6.32)	0.07–64.97	4.27 (4.12)	0.02–41.79	0.940 (0.069)	0.602–1.000	0.914 (0.044)	0.746–0.999
v5.0.0	−0.12 (2.27)	−26.63–5.94	4.70 (7.20)	0.02–89.48	3.39 (4.59)	0.01–55.11	0.963 (0.053)	0.635–1.000	0.925 (0.041)	0.669–0.999
Mac (OSX 10.5.8)										
v4.3.1 vs. v4.5.0	0.10 (1.36)	−2.66–7.79	5.02 (5.75)	0.00–64.79	3.05 (3.27)	0.00–34.76	0.958 (0.056)	0.683–1.000	0.940 (0.034)	0.771–1.000
v4.3.1 vs. v5.0.0	2.62 (7.64)	−29.76–33.51	9.36 (7.55)	1.58–85.62	8.75 (6.91)	1.30–59.52	0.812 (0.190)	0.000–0.999	0.857 (0.063)	0.565–0.973
v4.5.0 vs. v5.0.0	2.51 (7.81)	−35.16–34.05	9.31 (8.20)	1.62–96.70	8.76 (7.15)	1.35–63.98	0.816 (0.186)	0.000–0.999	0.858 (0.063)	0.536–0.973
HP										
v4.3.1 vs. v4.5.0	0.23 (1.63)	−8.22–8.20	5.24 (4.43)	0.00–37.02	3.26 (2.57)	0.00–17.35	0.953 (0.057)	0.586–1.000	0.934 (0.034)	0.825–1.000
v4.3.1 vs. v5.0.0	2.88 (7.32)	−20.82–33.18	9.53 (7.67)	2.18–85.06	8.87 (6.62)	1.52–53.75	0.806 (0.192)	0.581–0.972	0.856 (0.062)	0.581–0.972
v4.5.0 vs. v5.0.0	2.63 (6.95)	−12.89–33.24	9.30 (6.79)	2.23–69.90	8.59 (6.02)	1.48–45.06	0.814 (0.178)	0.000–0.999	0.859 (0.060)	0.628–0.972
MacOSX10.6.4 vs. 10.5.8										
v4.3.1	0.02 (1.67)	−5.71–12.09	5.55 (5.57)	0.00–55.76	3.79 (3.24)	0.00–24.18	0.948 (0.066)	0.645–1.000	0.920 (0.044)	0.764–1.000
v4.5.0	−0.09 (1.48)	−5.85–6.49	6.10 (6.08)	0.00–66.90	4.10 (3.70)	0.00–35.58	0.941 (0.066)	0.695–1.000	0.919 (0.044)	0.750–1.000
v5.0.0	−0.26 (2.15)	−25.61 (3.67)	4.50 (4.94)	0.00–52.96	3.28 (3.46)	0.00–36.59	0.962 (0.050)	0.701–1.000	0.925 (0.040)	0.719–1.000

a Coefficient of variation is the standard deviation of the signed differences relative to the average of the two volume measurements.

b ICC is the intra-class correlation coefficient.

c SI is the overlap value (Dice coefficient).

Note: the results for the tabulated volumes are almost identical.

**Table 4:** Summary of cortical thickness differences.

	Signed difference (%)		Coefficient of variation (%)^a^		Absolute difference (%)		ICC^b^	
Comparison	Mean (sd)	Range	Mean (sd)	Range	Mean (sd)	Range	Mean (sd)	Range
HP vs. Mac (OSX 10.5.8)								
v4.3.1	0.28 (0.48)	−0.56–2.02	2.04 (1.07)	0.70–5.27	1.58 (0.81)	0.53–3.96	0.956 (0.026)	0.895–0.995
v4.5.0	0.19 (0.62)	−1.87–2.24	2.02 (1.01)	0.81–5.64	1.56 (0.77)	0.61–3.77	0.956 (0.027)	0.885–0.992
v5.0.0	0.08 (0.34)	−0.89–0.93	1.89 (0.98)	0.70–5.44	1.45 (0.72)	0.55–4.14	0.958 (0.034)	0.804–0.994
Mac (OSX 10.5.8)								
v4.3.1 vs. v4.5.0	−0.05 (0.32)	−1.18–1.01	1.61 (0.83)	0.64–4.11	1.06 (0.53)	0.37–2.89	0.974 (0.016)	0.928–0.995
v4.3.1 vs. v5.0.0	−1.05 (1.51)	−6.89–2.84	3.14 (1.40)	1.36–7.57	2.80 (1.35)	1.22–7.69	0.869 (0.084)	0.555–0.971
v4.5.0 vs. v5.0.0	−1.00 (1.58)	−6.83–3.03	3.13 (1.42)	1.27–9.28	2.80 (1.35)	1.19–7.35	0.872 (0.083)	0.593–0.974
HP								
v4.3.1 vs. v4.5.0	0.04 (0.34)	−0.69–1.18	1.60 (0.88)	0.63–4.04	1.10 (0.60)	0.42–2.93	0.974 (0.017)	0.924–0.996
v4.3.1 vs. v5.0.0	−0.84 (1.27)	−5.17–2.68	3.21 (1.59)	1.23–10.39	2.69 (1.30)	1.10–7.07	0.870 (0.090)	0.569–0.978
v4.5.0 vs. v5.0.0	−0.88 (1.15)	−4.82–1.86	3.19 (1.48)	1.38–8.69	2.68 (1.21)	1.21–6.11	0.871 (0.086)	0.592–0.973
MacOSX10.6 vs. 10.5								
v4.3.1	0.28 (0.68)	−1.58–3.34	2.25 (1.77)	0.60–10.85	1.56 (0.92)	0.47–4.49	0.948 (0.058)	0.670–0.998
v4.5.0	0.12 (0.57)	−1.35–2.91	2.17 (1.51)	0.62–9.58	1.58 (0.86)	0.50–4.80	0.952 (0.042)	0.756–0.994
v5.0.0	0.07 (0.39)	−0.82–1.23	1.82 (1.00)	0.61–4.64	1.37 (0.73)	0.46–3.59	0.960 (0.038)	0.816–0.996

a Coefficient of variation is the standard deviation of the signed differences relative to the average of the two cortical thickness measurements.

b ICC is the intra-class correlation coefficient.

### Overlays of differences for version v4.3.1 vs. v5.0.0 (Mac)

**Figure 3:**
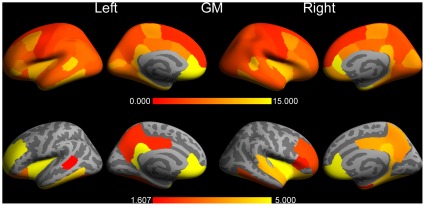
The differences in cortical grey matter volumes between FreeSurfer version v4.3.1 and v5.0.0 on a Mac (OSX 10.5). The upper row shows the left and right percentage absolute volume differences overlaid on the inflated respective hemispheres in lateral and medial views of an average brain (“fsaverage”). The differences are color coded between 0% and 15%, the full range was 2.1% (left precentral gyrus) - 24.9% (right rostral anterior cingulate cortex). The bottom row depicts the corresponding *p* values (expressed as −log10(*p*)) of the applied Student *t* test. The *p* values are color coded between the FDR level of 1.607 (*p* = 0.025) and 5.000 (*p* = 0.00001). The dark grey regions represent sulcal folds and the light grey regions represent gyral folds.

To zoom in on the largest differences observed and to show also the corresponding statistical significances, overlays were produced on the inflated pial surfaces of an average brain (so-called “fsaverage”, supplied by Freesurfer) for the comparison between Mac version v4.3.1 and v5.0.0. Figure 3 displays the results for the grey matter (GM) cortical structures. Again, these overlays demonstrate a non-uniformity in difference across the cortex. Note the highly significant (−log10(p)≥4; p≤0.0001) differences for the left and right frontal pole, left and right insula, left and right isthmus cingulate cortex, left and right medial orbital frontal cortex, left and right rostral anterior cingulate cortex, left inferior temporal gyrus, left rostral middle frontal cortex, left temporal pole, right fusiform gyrus, right lateral orbital frontal cortex, and right parahippocampal gyrus.

**Figure 4:**
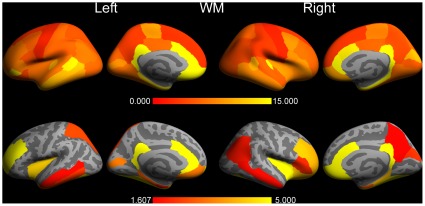
The same as **Figure 3**, but now for cortical white matter. The full range of the difference was 2.0% (left precentral gyrus) - 33.5% (right rostral anterior cingulate cortex).

The results for the cortical white matter (WM) structures (i.e., cortically associated gyral WM structures) are depicted in Figure 4. Although it may not be anatomically correct, these structures were overlaid also on the pial surfaces for visualisation purposes. The pattern of WM structures showing highly significant differences was rather similar to the pattern found for GM. Note that almost all of the largest differences are associated with the smallest p-values for both GM and WM.

**Figure 5:**
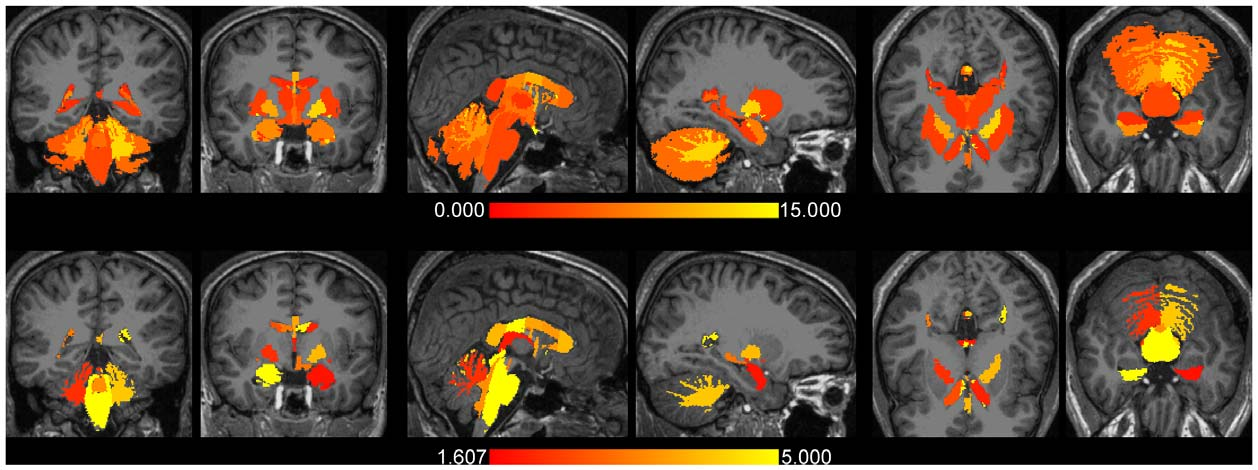
The differences in subcortical grey matter volumes between FreeSurfer version v4.3.1 and v5.0.0 on a Mac (OSX 10.5). The upper row shows the percentage absolute volume differences overlaid each time on two typical slices in coronal, saggital, and transversal views of a T1 scan of a subject, transformed to the MNI305 standard space. The differences are color coded between 0% and 15%, the full range was 2.3% (right lateral ventricle) - 59.5% (5^th^ ventricle). The bottom row depicts the corresponding *p* values (expressed as −log10(*p*)) of the applied Student *t* test. The *p* values are color coded between the FDR level of 1.607 (*p* = 0.025) and 5.000 (*p* = 0.00001).

For the subcortical structures we generated overlays on coronal, sagittal and transversal slices of T1 data of a single participant, transformed to the MNI305 standard space (Figure 5). Structures showing highly significant differences were the brain stem, the right amygdala, the right accumbens area, and the anterior, mid-posterior, and posterior partitions of the corpus callosum, left cerebellar white matter, and finally, the left lateral ventricle. Note that the fragmentation of some structures (e.g., cerebellum) is due to the application of a nearest neighbor interpolation in the transformation to the MNI305 template.

**Figure 6:**
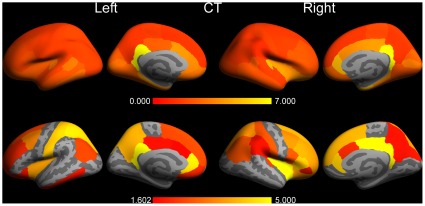
The same as **Figure 3**, but now for cortical thickness. The percentage absolute thickness differences is color coded between 0% and 7%, the full range was 1.2% (right supramarginal gyrus) - 7.7% (right isthmus cingulate cortex). A *p* value above 0.025 (−log10(p) = 1.602) was statistically significant after applying a FDR correction for multiple comparisons.

The pial surface overlays for the CT values are displayed in Figure 6. In this case highly significant differences were found for the structures left and right rostral anterior cingulate cortex, left and right isthmus cingulate cortex, left postcentral gyrus, left superior parietal cortex, left superior frontal gyrus, right insula, right pars triangularis, and right posterior cingulate cortex.

### Volume differences for specific set of structures

**Figure 7:**
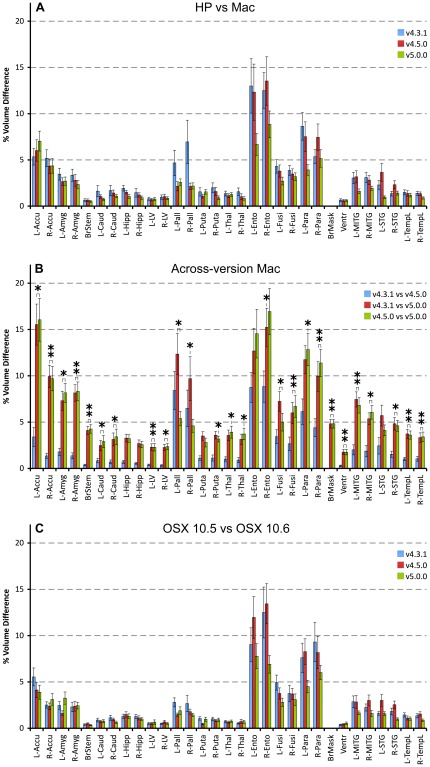
Effects of data processing conditions on the voxel volumes for a subsample of (sub)cortical structures. Panel A shows the detected percentage absolute differences between the results derived on a Mac and HP workstation for three different versions of FreeSurfer. Panel B depicts the differences between FreeSurfer v4.3.1 vs. v4.5.0, v4.3.1 vs. v5.0.0, and v4.5.0 vs. v5.0.0 for the Mac (OSX 10.5) (for HP these are very similar). Panel C displays the differences between OSX 10.6 and OSX 10.5. For comparison purposes the same vertical scale was used as in Figure 3 of Morey et al. [14] in which the same structures up to the left and right thalamus were considered. The significance is indicated at two levels: * : *p*<0.025 (the FDR level); ** : *p*≤0.0001. Abbreviations: L: left; R: right; Accu: accumbens; Amyg: amygdala; BrStem: brain stem; Caud: caudate; Hipp: hippocampus; LV: lateral ventricle; Pall: pallidum; Puta: putamen; Thal: thalamus; Ento: entorhinal cortex; Fusi: fusiform; Para: parahippocampal gyrus; BrMask: brain mask; Ventr: left+right lateral and inferior lateral ventricles; MITG: medial-inferior temporal gyrus; STG: superior temporal gyrus; TempL: temporal lobe.

The percentage voxel volume absolute differences for a number of structures frequently used in the literature (c.f., [5] and [14]) are shown in Figure 7. Examination of the contrasts Mac vs. HP (panel A) and OSX 10.5 vs. OSX 10.6 (panel C) illustrates the large volume differences for the left and right entorhinal cortex and left and right parahippocampal gyrus (up to about 10% and 5%, respectively). Also the left and right pallidum showed relatively large volume differences for v4.3.1. There was a slight tendency for a more robust segmentation in case of v5.0.0. The largest differences (up to about 17%) were observed between Mac v4.3.1 and v5.0.0 and between v4.5.0 and v5.0.0 (panel B). The structures found to be sensitive were: the left (especially) and right accumbens, left and right amygdala, entorhinal cortex, and parahippocampal gyrus. Moreover, large differences for the left and right pallidal volumes were present between v4.3.1 and v4.5.0 (about 7%), between v4.3.1 and v5.0.0 (about 10%), and between v4.5.0 and v5.0.0 (about 5%). The cross-version results for the HP were very similar. For almost all shown structures, the differences were significant in case of the v4.3.1/v4.5.0 vs. v5.0.0 contrast. See Figure S1 for similar graphs for CT.

### Additional results

Results regarding the determinant of the Talairach transformation matrix and variability under Mac OSX 10.6 can be found in Document S1 and Table S4.

## Discussion

In this study, an in-depth analysis was made of the performance of FreeSurfer under various data processing conditions, such as Mac and HP workstations and three versions, v4.3.1, v4.5.0 and v5.0.0. For this analysis, T1 scan data were used pertaining to a sample of 30 individuals participating in an ongoing longitudinal study. A number of experiments were conceived in order to gain insight into the variability of the results by repeating the data processing (“run”) for the same individual(s). To our knowledge no previous research of this type has been conducted, at least not for FreeSurfer. Significant differences in volume and cortical thickness were revealed across FreeSurfer versions. In addition, less pronounced differences were found between the Mac and HP workstations and between Mac OSX 10.5 and OSX 10.6.

### General findings

We first investigated if any differences would occur if runs were repeated on the same workstation using a single run or parallel runs. Since this did not reveal any differences, and thus not any interference between parallel running streams occurred, we could safely run as much as 8 pipelines simultaneously on both Mac and HP platforms. In this respect it may be stated that the FreeSurfer pipeline was properly designed. This considerably speeded up our workflow and made it possible to perform the other experiments in a reasonable amount of time (nevertheless about 300 days of computer time were consumed in the present study). Although all but one of the workstations had 16 GB RAM onboard, still some memory competition was being observed during parallel runs, adversely affecting the computation times by about 10–20%. It was also noted that version v5.0.0 was about 20–30% faster than the previous versions.

The other experiments conducted uncovered differences across workstations, FreeSurfer versions, and Mac OSX versions. Particularly large and significant differences in volume and cortical thickness were apparent between version v5.0.0 and earlier versions. In that case, about half of the 178 volume and 68 cortical thickness measurements were significant after FDR correction for multiple comparisons (without any correction almost all structures showed significant effects). Furthermore, the absolute differences were on average about 8.8% (for volumes) and 2.8% (for CT). It is beyond the scope of the present study to explore the origin of the cross-version differences in more detail. However, as the release notes describe changes to the correction for intensity non-uniformities and skull-strip stages in version v5.0.0, we anticipate that these changes present the main contributions to the observed cross-version effects.

### Comparison with results reported in the literature

The differences observed in the present study can be evaluated and put into the perspective of reliability and accuracy studies or studies (cross-sectional or longitudinal) on normal or pathological changes in cerebral morphology. Regarding volume reliability, Morey et al. [14] reported an average percentage absolute volume difference across scan sessions on the same scanner of 3.2±0.03% for nine subcortical structures (including brain stem and ventricles). For the same structures, we found differences between 2% (HP vs. Mac and Mac OSX 10.6 vs. 10.5 contrasts) and 5.5% (v4.3.1/v4.5.0 vs. v5.0.0 contrasts). Similar reproducibility errors were derived by Jovicich et al. [11]: 1.5–10.2% (absolute differences). Salat et al. [13] reported within-scanner reliabilities of white matter structures on the order of 5% (signed differences) with some exceptions as large as 29.7%. Note that these values are approximately comparable to our findings (Table 3). They even found the same large variabilities for the same structures (entorhinal cortex and frontal and temporal poles) as we did in comparing version v4.3.1/v4.5.0 with v5.0.0. The combined findings illustrate that these structures are especially susceptible to changing conditions, such as scan session and FreeSurfer version. Benedict et al. [12] reported within-scanner COVs between 0.7% and 7.7%. For the structures they considered, we derived COVs in the same range in case of the HP vs. Mac and OSX 10.6 vs. 10.5 comparisons. However, our cross-version COVs were about a factor of two larger and for the left and right amygdala even a factor of three to four larger.

Regarding cortical thickness reliability, Han et al. [9] reported a within-scanner variability (absolute difference) of global thickness <0.03 mm, corresponding to about 1.5%. This result is of the same order of magnitude as our findings (1.1–2.8%, see Table 4).

As for the accuracy of volume segmentation, Lehmann et al. [5] found a good correlation with manual segmentations for most of the structures they considered, with some exceptions (hippocampus, entorhinal cortex and fusiform gyrus) which they attributed to differences in delineation protocols. Since they did not present volume differences, only measures of overlap (Jaccard index) could be compared with our results. The range of Jaccard indices they reported was 0.05–0.89, corresponding to Dice indices in the range of 0.10–0.92, considerably smaller than our values which were all in excess of 0.70 for these structures. The accuracy of the hippocampus and amygdala was also studied by Morey et al. [4]: the percentage absolute volume differences with respect to manual were about 4.5% and 8.0%, respectively. These values are comparable to the cross-version differences derived in the present study (Figure 7).

Finally, the accuracy of cortical thickness was in one study better than 0.5 mm [7] and in another study better than 0.20 mm with a mean difference of 0.077 mm [6]. The latter value, corresponding to about 3.8%, is larger than the maximal mean difference of 2.8% we found.

Contrasting our results with structural changes in brain morphology due to pathology (Alzheimer or Huntington's disease) or neuropsychiatric disorders may be even more important. For instance, Lehmann et al. [5] reported on GM volume changes as a result of Alzheimer disease (AD) and semantic dementia (SD). They found absolute changes in volume with respect to controls in the range of 6–129% and 11–91%, respectively. Although these changes are about a factor of 10 larger than the largest differences we observed for the majority of structures, some structures are comparable in volume difference, such as the parahippocampal gyrus in AD (14%/28% (left/right) vs. 13%/11% reported here), superior temporal gyrus in AD (6%/6% vs. 4.1%/4.6%) and whole brain (4–11% vs. 4.8%). Notice that atrophy of the parahippocampal gyrus has recently been suggested as an early biomarker of AD [32]. Regional white matter volume differences between normal aging and AD were estimated at between 0.2% and 25.9% (Salat et al. 2009), again comparable to the differences we derived (Table 3). With respect to cortical thickness, Dickerson et al. [33] reported cortical thinning between 2.3% and 13.6% in AD patients compared to non-demented older controls. Rosas et al. [6],[34] studied the impact of Huntington's disease on cortical thinning. They derived differences between 5% in early stages and up to 30% in late stages. Finally, comparing patients suffering from schizophrenia with healthy controls, cortical thickness differences were in the range of −6.7–5.3% [7]. In our comparisons we found differences between 0.4% and 7% (Table 4), approximately of the same order of magnitude as all these reported effect sizes.

Summarising all the above comparisons, we can draw the conclusion that effect sizes resulting from differences in data processing conditions are rather similar to reliability and accuracy measurements previously reported in the literature. Therefore, our results suggest that these reliability and accuracy measurements depend on specific processing conditions, especially the version of FreeSurfer that was used. Moreover, the effect sizes we derived are more or less of the same order of magnitude as those reported in case-control comparisons in neuropsychiatric illness. The consequence is that the power of such studies may be compromised by changing the data processing conditions. In additon, the observed effects may have profound implications for longitudinal studies: if processing conditions have changed it is recommended to re-run the analysis and to absorb the computational cost. Our findings not only support the recommendations issued explicitly by the FreeSurfer developers over the last years not to mix FreeSurfer versions, platforms, and OS versions, but also validate these in terms of quantification of associated effect sizes. It should be noted in this context that not every upgrade in OS or FreeSurfer will affect the outcome of a study. Minor upgrades in the OS (e.g., from OSX 10.6.4 to OSX10.6.5) usually bring security patches in order to maintain a safe computing environment and they will not lead to different results. Also some minor upgrades in FreeSurfer (e.g., from v4.3.0 to v4.3.1) are necessary to fix bugs specific to some area not related to the research and they can be done without affecting the results. However, a meticulous inspection of the accompanying release notes is mandatory to be sure of a safe upgrade.

### Other effects

A discussion on the determinant of the Talairach transformation matrix, Mac vs. HP inconsistencies, the effects of Mac OS version, and variability under Mac OSX 10.6 can be found in Document S1.

### Limitations

One limitation of the present study is that no direct analysis is made of how the processing conditions evaluated may affect the accuracy of the volume and cortical thickness measurements. It should be noted that a difference between two FreeSurfer versions does not intrinsically imply a difference in accuracy. An accuracy assessment requires manual segmentations and/or histological measurements. Although this is beyond the scope of our study, it may be a suggestion for future research.

Another limitation of the study may concern the participant sample. It comprised 10 healthy controls, 10 patients with psychotic disorder and 10 siblings of patients with psychotic disorder. A total of 30 individuals may be rather low, however, it represented a compromise between enough power and excessively long processing times. The sample is not representative in comparison with other studies including, for example, patients with Alzheimer's or Huntington's disease. However, it has been shown that FreeSurfer reliably captures subtle morphological and pathological changes in the brain, demonstrating that its performance is independent of the cerebral morphology. Therefore, the results presented here may be considered indicative of expected variabilities in FreeSurfer.

Finally, there may be other variables affecting the volume and cortical thickness measurements, for example CPU type and bits mode (32 or 64 bits) of the OS. The FreeSurfer users install it on various computer workstations, not only MAC, HP workstation, but also Dell, cluster supercomputers, personally assembled computers. So, an almost infinite mixture of CPU types and operating systems exists. It is impracticable to test FreeSurfer on all possible combinations of hardware and software. The type of workstation considered in the present study is just one variable among various hardware environments affecting the results of FreeSurfer.

### Conclusions

The general conclusion from the present study and the practical advice it occasions is that users of FreeSurfer should exercise caution and restraint before applying a major upgrade in either the FreeSurfer (in particular) or OS version or to switch to a different type of workstation in the context of an ongoing study. This may be a truism and consistent with sound methodology for scientific experimentation and therefore a matter of common sense. However, the numerous questions about this issue posted by the user community seem to demonstrate the opposite and the results presented here reliably quantify the possible consequences. The message of caution applies not only to FreeSurfer but likely may be generalised to other intricate processing packages in the field of neuroimaging. The packages become more and more complex and therefore it is difficult to keep a check on propagation effects resulting from (small) modifications regarding one of the underlying algorithms.

An additional message inferred from the present study is that authors reporting on results obtained with FreeSurfer are highly recommended to provide not only the version of FreeSurfer that was used but also details on the OS version and workstation.

Finally, given the large and significant differences between the latest version v5.0.0 and earlier versions, it is concluded that an assessment of the accuracy of FreeSurfer is desirable.

## Supporting Information

Document S1 This document is an extension to some of the sections of the main document. In “Supplementary materials and methods” more details are provided on FreeSurfer and statistical measures used. Of special notice is the randomness in a few algorithms in the processing pipeline of FreeSurfer version v4.3.1 only. These are all utilities operating on surfaces and therefore solely affecting cortical structures. The section “Supplementary results” presents results regarding the determinant of the Talairach transformation matrix and the variability under Mac OSX 10.6 for FreeSurfer versions v4.3.1 and v4.5.0. In addition, the effects of randomness are presented. In the section “Supplementary discussion” the above results are discussed and furthermore, the differences between Mac and HP and between OSX 10.6 and 10.5. Finally, the “Appendix” provides details on the composite volumes listed in Lehmann et al., 2010.
(PDF)

Figure S1 **Effects of data processing conditions on the cortical thickness for 68 cortical structures.** Panel A shows the detected percentage absolute differences between the results derived on a Macintosh and HP workstation for three different versions of FreeSurfer. Panel B depicts the differences between FreeSurfer v4.3.1 vs. v4.5.0, v4.3.1 vs. v5.0.0, and v4.5.0 vs. v5.0.0 for the Macintosh (for HP these are very similar). Panel C displays the differences between OSX 10.6 and OSX 10.5. The left/right column refers to the left/right hemisphere. The structures labeled along the X-axes in Panel A are numbered in order to label the corresponding X-axes in the other two panels. The significance is indicated at two levels: * : *p*<0.025 (the FDR level, cf. Table 2); ** : *p*≤0.0001.
(TIF)

Table S1 Detailed statistics of the voxel volume measurements.
(XLS)

Table S2 Detailed statistics of the tabulated volume measurements.
(XLS)

Table S3 Detailed statistics of the cortical thickness measurements.
(XLS)

Table S4 Effects of randomness on volume and cortical thickness measurements.
(PDF)

## References

[1] Fischl B (2012). FreeSurfer.. Neuroimage.

[2] Desikan RS, Segonne F, Fischl B, Quinn BT, Dickerson BC (2006). An automated labeling system for subdividing the human cerebral cortex on MRI scans into gyral based regions of interest.. Neuroimage.

[3] Tae WS, Kim SS, Lee KU, Nam EC, Kim KW (2008). Validation of hippocampal volumes measured using a manual method and two automated methods (FreeSurfer and IBASPM) in chronic major depressive disorder.. Neuroradiology.

[4] Morey RA, Petty CM, Xu Y, Hayes JP, Wagner HR (2009). A comparison of automated segmentation and manual tracing for quantifying hippocampal and amygdala volumes.. Neuroimage.

[5] Lehmann M, Douiri A, Kim LG, Modat M, Chan D (2010). Atrophy patterns in Alzheimer's disease and semantic dementia: a comparison of FreeSurfer and manual volumetric measurements.. Neuroimage.

[6] Rosas HD, Liu AK, Hersch S, Glessner M, Ferrante RJ (2002). Regional and progressive thinning of the cortical ribbon in Huntington's disease.. Neurology.

[7] Kuperberg GR, Broome MR, McGuire PK, David AS, Eddy M (2003). Regionally localized thinning of the cerebral cortex in schizophrenia.. Arch Gen Psychiatry.

[8] Salat DH, Buckner RL, Snyder AZ, Greve DN, Desikan RS (2004). Thinning of the cerebral cortex in aging.. Cereb Cortex.

[9] Han X, Jovicich J, Salat D, van der Kouwe A, Quinn B (2006). Reliability of MRI-derived measurements of human cerebral cortical thickness: the effects of field strength, scanner upgrade and manufacturer.. Neuroimage.

[10] Dickerson BC, Fenstermacher E, Salat DH, Wolk DA, Maguire RP (2008). Detection of cortical thickness correlates of cognitive performance: Reliability across MRI scan sessions, scanners, and field strengths.. Neuroimage.

[11] Jovicich J, Czanner S, Han X, Salat D, van der Kouwe A (2009). MRI-derived measurements of human subcortical, ventricular and intracranial brain volumes: Reliability effects of scan sessions, acquisition sequences, data analyses, scanner upgrade, scanner vendors and field strengths.. Neuroimage.

[12] Benedict RH, Ramasamy D, Munschauer F, Weinstock-Guttman B, Zivadinov R (2009). Memory impairment in multiple sclerosis: correlation with deep grey matter and mesial temporal atrophy.. J Neurol Neurosurg Psychiatry.

[13] Salat DH, Greve DN, Pacheco JL, Quinn BT, Helmer KG (2009). Regional white matter volume differences in nondemented aging and Alzheimer's disease.. Neuroimage.

[14] Morey RA, Selgrade ES, Wagner HR, Huettel SA, Wang L (2010). Scan-rescan reliability of subcortical brain volumes derived from automated segmentation.. Hum Brain Mapp.

[15] Nylenna M, Riis P (1991). Identification of patients in medical publications: need for informed consent.. Bmj.

[16] Habets P, Marcelis M, Gronenschild E, Drukker M, van Os J (2011). Reduced cortical thickness as an outcome of differential sensitivity to environmental risks in schizophrenia.. Biol Psychiatry.

[17] Dale AM, Fischl B, Sereno MI (1999). Cortical surface-based analysis. I. Segmentation and surface reconstruction.. Neuroimage.

[18] Fischl B, Sereno MI, Dale AM (1999). Cortical surface-based analysis. II: Inflation, flattening, and a surface-based coordinate system.. Neuroimage.

[19] Sled JG, Zijdenbos AP, Evans AC (1998). A nonparametric method for automatic correction of intensity nonuniformity in MRI data.. IEEE Trans Med Imag.

[20] Segonne F, Dale AM, Busa E, Glessner M, Salat D (2004). A hybrid approach to the skull stripping problem in MRI.. Neuroimage.

[21] Fischl B, Salat DH, Busa E, Albert M, Dieterich M (2002). Whole brain segmentation: automated labeling of neuroanatomical structures in the human brain.. Neuron.

[22] Fischl B, Salat DH, van der Kouwe AJ, Makris N, Segonne F (2004). Sequence-independent segmentation of magnetic resonance images.. Neuroimage.

[23] Fischl B, Liu A, Dale AM (2001). Automated manifold surgery: constructing geometrically accurate and topologically correct models of the human cerebral cortex.. IEEE Trans Med Imaging.

[24] Segonne F, Pacheco J, Fischl B (2007). Geometrically accurate topology-correction of cortical surfaces using nonseparating loops.. IEEE Trans Med Imaging.

[25] Dale AM, Sereno MI (1993). Improved Localizadon of Cortical Activity by Combining EEG and MEG with MRI Cortical Surface Reconstruction: A Linear Approach.. J Cogn Neurosci.

[26] Fischl B, Sereno MI, Tootell RB, Dale AM (1999). High-resolution intersubject averaging and a coordinate system for the cortical surface.. Hum Brain Mapp.

[27] Fischl B, van der Kouwe A, Destrieux C, Halgren E, Segonne F (2004). Automatically parcellating the human cerebral cortex.. Cereb Cortex.

[28] Fischl B, Dale AM (2000). Measuring the thickness of the human cerebral cortex from magnetic resonance images.. Proc Natl Acad Sci U S A.

[29] Hammers A, Heckemann R, Koepp MJ, Duncan JS, Hajnal JV (2007). Automatic detection and quantification of hippocampal atrophy on MRI in temporal lobe epilepsy: a proof-of-principle study.. Neuroimage.

[30] Shrout PE, Fleiss JL (1979). Intraclass correlations: uses in assessing rater reliability.. Psychological Bulletin.

[31] Benjamini Y, Hochberg Y (1995). Controlling the false discovery rate: a practical and powerful approach to multiple testing.. J R Stat Soc, B Methodol.

[32] Echavarri C, Aalten P, Uylings HB, Jacobs HI, Visser PJ (2010). Atrophy in the parahippocampal gyrus as an early biomarker of Alzheimer's disease.. Brain Struct Funct.

[33] Dickerson BC, Bakkour A, Salat DH, Feczko E, Pacheco J (2009). The cortical signature of Alzheimer's disease: regionally specific cortical thinning relates to symptom severity in very mild to mild AD dementia and is detectable in asymptomatic amyloid-positive individuals.. Cereb Cortex.

[34] Rosas HD, Salat DH, Lee SY, Zaleta AK, Pappu V (2008). Cerebral cortex and the clinical expression of Huntington's disease: complexity and heterogeneity.. Brain.

