# Supplementary material for: The Effects of FreeSurfer Version, Workstation Type, and Macintosh Operating System Version on Anatomical Volume and Cortical Thickness Measurements
Source: PLoS One. 2012 Jun 1;7(6):e38234. doi: 10.1371/journal.pone.0038234 (PMC3365894; doi:10.1371/journal.pone.0038234)
Supplement: Document S1 — This document is an extension to some of the sections of the main document. In “Supplementary materials and methods” more details are provided on FreeSurfer and statistical measures used. Of special notice is the randomness in a few algorithms in the processing pipeline of FreeSurfer version v4.3.1 only. These are all utilities operating on surfaces and therefore solely affecting cortical structures. The section “Supplementary results” presents results regarding the determinant of the Talairach transformation matrix and the variability under Mac OSX 10.6 for FreeSurfer versions v4.3.1 and v4.5.0. In addition, the effects of randomness are presented. In the section “Supplementary discussion” the above results are discussed and furthermore, the differences between Mac and HP and between OSX 10.6 and 10.5. Finally, the “Appendix” provides details on the composite volumes listed in Lehmann et al., 2010. (PDF) [file pone.0038234.s001.pdf]

The Effects of FreeSurfer Version, Workstation Type, and Macintosh Operating System Version on Anatomical Volume and Cortical Thickness Measurements

- Supplement -

Ed H.B.M. Gronenschild, Petra Habets, Heidi I.L. Jacobs, Ron Mengelers, Nico Rozendaal, Jim van Os, Machteld Marcelis.

Supplementary materials and methods

FreeSurfer

The FreeSurfer analysis pipeline comprises two main processing streams, a volume-based stream and a surface-based stream. The volume-based stream is designed to assign a neuroanatomical label to each voxel, whereas the surface-based stream is developed to derive the white and pial surfaces [1,2]. The volume-based stream involves correction for intensity non-uniformity in the MRI data [3], affine transformation to the MNI305 template, intensity normalisation, removal of non-brain tissue [4], linear and non-linear transformations to a probabilistic brain atlas, and finally, labeling of cortical and subcortical structures (both white and grey matter) [5,6]. The surface-based stream starts with separating the two hemispheres, followed by tesselation of the grey – white matter boundary [7], topology correction [8], and surface deformation to follow the intensity gradients between white and grey matter (called white surface) as well as between grey matter and CSF (called pial surface) [1,9]. Next, the surfaces are inflated to a sphere [2] and registered to a spherical atlas [10] after which a parcellation is performed of the cerebral cortex into units based on the gyral and sulcal structure [11,12]. An array of surface based derivatives is created, including cortical thickness (CT) and maps of curvature and sulcal depth. The cortical thickness value at each vertex on the white surface is calculated as the avarage of the distance from a point on the white surface to the closest point on the pial surface and from that point back to the closest point on the white surface [13].

The results of FreeSurfer are presented in the form of voxel data (so-called ".mgz" files), surfaces, CT maps, curvature maps, sulcal depth maps, and tables (so-called ".stats" files). By virtue of a so-called segmentation ID (segID), a one-to-one correspondence exists between the volumes which can be derived from the labeled voxel data and the volumes listed

in the tables. The difference is that the tabulated volumes are more accurate because they are corrected for partial volume effects. The following voxel data and tabulated data were used in our analysis:

- aseg.mgz (for 43 subcortical volumes, segIDs in the range 1 - 255)
- wmparc.mgz (for 68 cortical volumes, segIDs 3001 – 3034 and 4001 – 4034)
- aseg.stats (for 43 subcortical volumes, segIDs 1 – 255, and some total brain related volumes)
- rh/lh.aparc.stats (for 68 cortical volumes and 68 CT values, segIDs 1001 – 1035 and 2001 - 2035)
- brainmask.mgz (the skull-stripped brain)

In addition, the table aseg.stats lists four volumes related to the total brain, such as for instance “Brain Mask Volume”. These tabulated volumes do not have a direct counterpart in the voxel data. Of special notice are the tabulated volumes called “Brain Segmentation Volume” and “Brain Segmentation Volume Without Ventricles” in version v4.3.1 and v4.5.0. The counterpart volume for the first could be derived by summing up all labeled voxels in the aseg.mgz file and the counterpart volume for the latter was obtained by subtracting from the first volume the volume of the labeled ventricles in the aseg.mgz file. In version v5.0.0, two new tabulated volumes were added: “Total gray matter volume” and “Total white matter volume". Unfortunately, no corresponding voxel volumes could be defined for these. Finally, the counterpart of the tabulated volume called “Brain Mask Volume” (BrMask) was derived by counting all voxels within brainmask.mgz.

As described above, in the volume-based stream a linear transformation to the MNI305 template is computed. This 12-parameter affine transformation matrix is saved as a file called “talairach.xfm”. Apart from the fact that a number of downstream tools use Talairach coordinates as seed points, the determinant of this transformation matrix is used to obtain the “Intracranial Volume” as listed in aseg.stats table. For these reasons, the determinant of this matrix was computed for further analysis.

A few algorithms in the processing pipeline are initialised by a pseudo-random number generator using the date and time as seed. These are all utilities operating on surfaces, i.e., collections of vertices and polygons, and therefore solely affecting cortical structures. Randomness enters into these algorithms only in deciding which vertex to begin operating at, as it is arbitrary (Nick Schmansky, Athinoula A. Martinos Center for Biomedical Imaging, Harvard, Boston, private communication). This may produce different results for repeated processing. The default "randomness" mode was changed by the authors of FreeSurfer to “norandomness” as of version v4.5.0, meaning that the involved tools are seeded with the

same fixed value such that repeated processing will produce identical results. We tried to obtain an accurate estimate of the variability due to randomness (FreeSurfer version v4.3.1 on a Mac with OSX 10.6.5) by repeating a run 150 times for three individuals (one of each group) with the randomness flag set. Naturally, in the Experiments 1 through 4, described in the main paper, the "norandomness" mode was used.

Statistical Measures

Several statistical measures were employed to quantify the effects studied. The most commonly used indicator is the percentage difference (ΔM) between the two repeated measurements (M_1 and M_2), defined as:

$$\Delta M = \frac{|M_1 - M_2|}{\left(\frac{M_1 + M_2}{2}\right)} x100, \tag{1}$$

where M is either the volume (V) or CT of a structure. In addition, means and standard deviations of M_1, M_2, $M_2 - M_1$ and $|M_2 - M_1|$ were obtained from the data. Moreover, the coefficient of variation (COV), defined as the percentage standard deviation relative to the mean, was computed for $M_2 - M_1$.

Since we are also dealing with voxel data, we computed the measure of spatial overlap of a structure, defined as:

$$SI = \frac{V(S_1 \cap S_2)}{\left(\frac{V(S_1) + V(S_2)}{2}\right)}, \tag{2}$$

where $V(S)$ is the volume of structure S. This measure is also known as similarity index (SI) or Dice coefficient. Its range is between 0 (no overlap) and 1 (complete overlap). Likewise, we computed the Jaccard index, a second measure of spatial overlap that is frequently used (also known as overlap ratio (OR); OR can be derived from SI by the formula $OR = SI / (2 - SI)$ and conversely, $SI = 2\ OR / (1+OR)$ [14]). Measures of overlap are especially useful because the spatial properties (location and shape) of a structure are taken into account.

As described above, the variability due to randomness was assessed for version v4.3.1 by repeating the runs 150 times for three individuals. From the data, descriptive statistics were extracted, such as mean, standard deviation, median, minimum and maximum values. In addition, the results were compared to those from the corresponding runs with the no randomness flag set.

Supplementary results

Determinant of Talairach transformation matrix

The determinant of the Talairach transformation matrix (and thus the intracranial volume) diverged between the HP and Mac platforms for all versions (not significant, $p \approx 0.1$) and between either version v4.3.1 or v4.5.0 on the one hand and version v5.0.0 on the other hand for both platforms ($p < 0.0001$). Between Mac OSX 10.5 and Mac OSX 10.6 no differences were observed. More details can be found in Table S2.

Variability under Mac OSX 10. 6 for FreeSurfer versions v4.3.1 and v4.5.0

During one of the described experiments we noticed an unexpected variability of the results in two individuals under Mac OSX 10.6 only. In a post-hoc attempt to understand this, a small experiment was performed in which we repeated the runs 10 times under these data processing conditions. For v4.3.1, the COV was between -2.7% and 4.6% in case of the volumes and between -8.1% and 6.2% in case of CT. For v4.5.0, this variability occurred in only one participant and was substantially less: between -0.01% and 0.03% (volumes) and between -0.24% and 0.28% (CT). For v5.0.0, no variations could be detected at all. With respect to the volumes, the GM structures right lingual gyrus and right parahippocampal gyrus were involved, as were the WM structures left and right corpus callosum, right banks superior temporal sulcus, right isthmus and posterior cingulate cortex, and right lingual gyrus. For CT, the affected structures were right banks superior temporal sulcus, right isthmus cingulate cortex, right lingual gyrus, and right parahippocampal gyrus.

Effect of randomness

The variability of the 150 voxel volume and CT measurements due to randomness is summarised in Table S4. The COV, averaged over all structures and the three individuals, was 2.43 ± 2.52% (0.28 - 20.37%) for the volumes and 0.66 ± 0.63% (0.10 - 3.88%) for CT. The random values turned out to be not normally distributed and the mean value for either

volume or CT of a structure was not equal to the corresponding non-random value. This bias, expressed as percentage difference of the mean value with respect to the corresponding non-random value (taken relative to this latter value) and averaged over all structures and the three individuals, was 0.12 ± 3.29% with a range of -14.93% to 20.91% for the volume and -0.11 ± 0.94% with a range of -6.81% to 2.96% for CT. These differences were not only highly non-uniform across the cortex but also across the three individuals.

Supplementary discussion

Determinant of Talairach transformation matrix and Mac vs. HP inconsistencies

The determinant of the matrix describing the linear transformation to the MNI305 template was found to depend on the particular workstation and FreeSurfer version that was used. In an attempt to diagnose these variabilities, intermediate results in the FreeSurfer pipeline were monitored. The computation of the transformation matrix occurs early in the FreeSurfer pipeline; in fact, it is the second step executed just after the correction for intensity non-uniformities ("nu-correction"). In the comparison of HP vs. Mac, it was found that the computation of the matrix was the first instance in the pipeline where inconsistencies did arise. As each platform has its own implementation of the mathematical libraries (32 bits version for Mac (UNIX) and 64 bits version for HP (LINUX)) we assume that the source of these inconsistencies can be traced back to these implementation differences. This may explain (in part) the observed differences in the transformation matrix and final results between both platforms. If this were true then we would always expect the nu-correction as the first occurrence of inconsistencies between different platforms since it heavily relies on mathematical operations [3]. However, this is not what we discerned and therefore the exact nature of the transformation matrix inconsistencies remains unclear.

Whilst monitoring intermediate results we noticed that between v5.0.0 and earlier versions, the very first step in the pipeline, the nu-correction, already diverged. This may be attributed to changes to the algorithm as reported in the release notes and this in turn most probably accounted for the observed significant differences in the transformation matrix.

Effects of Mac OS version

Similarly, the differences between OS X 10.6 and OS X 10.5 could be caused by implementation effects of the mathematical libraries. Whereas OS X 10.5 is running in 32 bits, OS X 10.6 can run in 64 bits mode (although we always used 32 bits mode). However,

we did not detect any differences in the first steps in the pipeline, up to the intensity normalisation. The fact that the differences between the two OSX versions were almost identical to those between Mac and HP is suggestive of a common origin. It is beyond the scope of the present study to locate the subsequent step in the pipeline after the intensity normalisation that evoked the observed inconsistencies.

Variability under Mac OSX 10.6 for FreeSurfer versions v4.3.1 and v4.5.0

On the same platform and using the same FreeSurfer and OS version the results produced by FreeSurfer were perfectly reproducible. However, a serendipitous and unexpected discovery was the variability of the results in case of versions v4.3.1 and v4.5.0 used under Mac OSX 10.6 only and solely occurring in two out of 30 individuals. The variabilities in v4.5.0 were substantially smaller than those in v4.3.1 and their existence in v5.0.0 can be ruled out for certain. Only a few structures were affected, confined to almost exclusively the right hemisphere. These findings showed evidence of an unstable component in the pipeline only manifest under OSX 10.6 and not OSX 10.5, the origin of which remains obscure. Although the observed effects are relatively small, it is something to consider as a potentially important nuisance factor in studies. In future updates of FreeSurfer, these initially small variabilities may progressively grow to substantial magnitude, given the complex algorithms with multistage data processing and probabilistic computations, and may even reach the same order of magnitude as the other variabilities assessed in the present study. For this reason this issue deserves further attention and investigation by the developers.

Effects of randomness

Many users of FreeSurfer may have been ignorant of the presence of randomness in the processing pipeline, in particular for versions earlier than v4.5.0, and thus may have assumed that the results would be 100% reproducible, as we did initially. The main reason is the fact that this was not well documented on the FreeSurfer website. It was found that the effects due to randomness were not only highly non-uniform across the cortex but also across the three individuals studied, suggesting a large dependence on the cerebral morphology. Comparing the derived effects with reported variabilities in test-retest studies we may conclude that part of these variabilities may be attributed to random effects. A further consequence of randomness is that the power of a number of cross-sectional and longitudinal studies may have been overestimated since random effects are of the same order as the reported effect sizes and in addition, may depend on the particular cerebral morphology in each individual. It is for this reason that we fully support the decision by the FreeSurfer

authors to change the default mode to non-randomness as of version v4.5.0, although on average this introduces a bias with respect to the random results.

Appendix

The merged volumes listed in Lehmann et al., 2010 were composed of the following FreeSurfer segIDs and names (left and right hemisphere, respectively):

- Medial-inferior temporal gyrus (left and right)

  1009 (ctx-lh-inferiortemporal) / 2009 (ctx-rh-inferiortemporal)

  3009 (wm-lh-inferiortemporal) / 4009 (wm-rh-inferiortemporal)

  1015 (ctx-lh-middletemporal) / 2015 (ctx-rh-middletemporal)

  3015 (wm-lh-middletemporal) / 4015 (wm-rh-middletemporal)

- Superior temporal gyrus (left and right)

  1030 (ctx-lh-superiortemporal) / 2030 (ctx-rh-superiortemporal)

  3030 (wm-lh-superiortemporal) / 4030 (wm-rh-superiortemporal)

- Temporal lobe (left and right)

  17 (Left-Hippocampus) / 53 (Right-Hippocampus)

  18 (Left Amygdala) / 54 (Right-Amygdala)

  1006 (ctx-lh-entorhinal) / 2006 (ctx-rh-entorhinal)

  3006 (wm-lh-entorhinal) / 4006 (wm-rh-entorhinal)

  1007 (ctx-lh-fusiform) / 2007 (ctx-rh-fusiform)

  3007 (wm-lh-fusiform) / 4007 (wm-rh-fusiform)

  1009 (ctx-lh-inferiortemporal) / 2009 (ctx-rh-inferiortemporal)

  3009 (wm-lh-inferiortemporal) / 4009 (wm-rh-inferiortemporal)

  1015 (ctx-lh-middletemporal) / 2015 (ctx-rh-middletemporal)

  3015 (wm-lh-middletemporal) / 4015 (wm-rh-middletemporal)

  1016 (ctx-lh-parahippocampal) / 2016 (ctx-rh-parahippocampal)

  3016 (wm-lh-parahippocampal) / 4016 (wm-rh-parahippocampal)

  1030 (ctx-lh-superiortemporal) / 2030 (ctx-rh-superiortemporal)

  3030 (wm-lh-superiortemporal) / 4030 (wm-rh-superiortemporal)

  1033 (ctx-lh-temporalpole) / 2033 (ctx-rh-temporalpole)

  3033 (wm-lh-temporalpole) / 4033 (wm-rh-temporalpole)

  1034 (ctx-lh-transversetemporal) / 2034 (ctx-rh-transversetemporal)

  3034 (wm-lh-transversetemporal) / 4034 (wm-rh-transversetemporal)

- Ventricles

  4 (Left-Lateral-Ventricle) + 43 (Right-Lateral-Ventricle)

  5 (Left-Inf-Lat-Ven) + 44 (Right-Inf-Lat-Ven)

References

1. Dale AM, Fischl B, Sereno MI (1999) Cortical surface-based analysis. I. Segmentation and surface reconstruction. Neuroimage 9: 179-194.
2. Fischl B, Sereno MI, Dale AM (1999) Cortical surface-based analysis. II: Inflation, flattening, and a surface-based coordinate system. Neuroimage 9: 195-207.
3. Sled JG, Zijdenbos AP, Evans AC (1998) A nonparametric method for automatic correction of intensity nonuniformity in MRI data. IEEE Trans Med Imag 17: 87-97.
4. Segonne F, Dale AM, Busa E, Glessner M, Salat D, et al. (2004) A hybrid approach to the skull stripping problem in MRI. Neuroimage 22: 1060-1075.
5. Fischl B, Salat DH, Busa E, Albert M, Dieterich M, et al. (2002) Whole brain segmentation: automated labeling of neuroanatomical structures in the human brain. Neuron 33: 341-355.
6. Fischl B, Salat DH, van der Kouwe AJ, Makris N, Segonne F, et al. (2004) Sequence-independent segmentation of magnetic resonance images. Neuroimage 23 Suppl 1: S69-84.
7. Fischl B, Liu A, Dale AM (2001) Automated manifold surgery: constructing geometrically accurate and topologically correct models of the human cerebral cortex. IEEE Trans Med Imaging 20: 70-80.
8. Segonne F, Pacheco J, Fischl B (2007) Geometrically accurate topology-correction of cortical surfaces using nonseparating loops. IEEE Trans Med Imaging 26: 518-529.
9. Dale AM, Sereno MI (1993) Improved Localizadon of Cortical Activity by Combining EEG and MEG with MRI Cortical Surface Reconstruction: A Linear Approach. J Cogn Neurosci 5: 162-176.
10. Fischl B, Sereno MI, Tootell RB, Dale AM (1999) High-resolution intersubject averaging and a coordinate system for the cortical surface. Hum Brain Mapp 8: 272-284.
11. Desikan RS, Segonne F, Fischl B, Quinn BT, Dickerson BC, et al. (2006) An automated labeling system for subdividing the human cerebral cortex on MRI scans into gyral based regions of interest. Neuroimage 31: 968-980.
12. Fischl B, van der Kouwe A, Destrieux C, Halgren E, Segonne F, et al. (2004) Automatically parcellating the human cerebral cortex. Cereb Cortex 14: 11-22.
13. Fischl B, Dale AM (2000) Measuring the thickness of the human cerebral cortex from magnetic resonance images. Proc Natl Acad Sci U S A 97: 11050-11055.

14. Hammers A, Heckemann R, Koepp MJ, Duncan JS, Hajnal JV, et al. (2007) Automatic detection and quantification of hippocampal atrophy on MRI in temporal lobe epilepsy: a proof-of-principle study. Neuroimage 36: 38-47.
