# Supplementary figures and images for: The Effects of FreeSurfer Version, Workstation Type, and Macintosh Operating System Version on Anatomical Volume and Cortical Thickness Measurements
Source: PLoS One. 2012 Jun 1;7(6):e38234. doi: 10.1371/journal.pone.0038234 (PMC3365894; doi:10.1371/journal.pone.0038234)

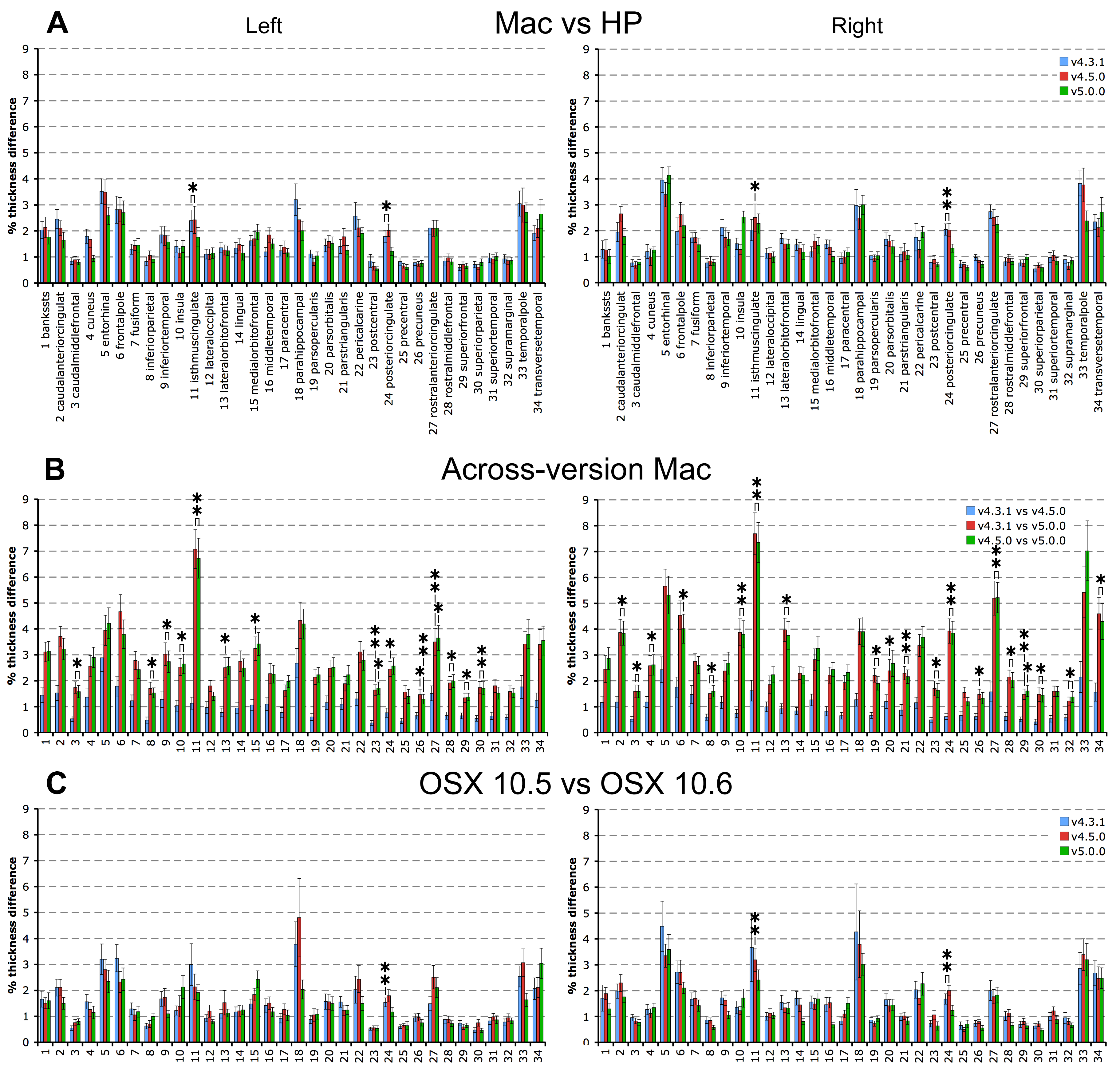

Supplement: Figure S1 — Effects of data processing conditions on the cortical thickness for 68 cortical structures. Panel A shows the detected percentage absolute differences between the results derived on a Macintosh and HP workstation for three different versions of FreeSurfer. Panel B depicts the differences between FreeSurfer v4.3.1 vs. v4.5.0, v4.3.1 vs. v5.0.0, and v4.5.0 vs. v5.0.0 for the Macintosh (for HP these are very similar). Panel C displays the differences between OSX 10.6 and OSX 10.5. The left/right column refers to the left/right hemisphere. The structures labeled along the X-axes in Panel A are numbered in order to label the corresponding X-axes in the other two panels. The significance is indicated at two levels: * : p<0.025 (the FDR level, cf. Table 2); ** : p≤0.0001. (TIF) [file pone.0038234.s002.tif]
