# Supplementary material for: The Effects of FreeSurfer Version, Workstation Type, and Macintosh Operating System Version on Anatomical Volume and Cortical Thickness Measurements
Source: PLoS One. 2012 Jun 1;7(6):e38234. doi: 10.1371/journal.pone.0038234 (PMC3365894; doi:10.1371/journal.pone.0038234)
Supplement: Table S4 — Effects of randomness on volume and cortical thickness measurements. (PDF) [file pone.0038234.s006.pdf]

Table S4. Effects of randomness on volume and cortical thickness measurements

Participant	Coefficient of variation (%)[a]		Skewness		Difference (%)[b]	
	Mean (sd)	Range	Mean (sd)	Range	Mean (sd)	Range
Volume						
1	3.06 (3.34)	0.33 - 20.37	0.16 (1.69)	-5.90 - 7.35	-0.23 (3.41)	-14.93 - 13.46
2	2.21 (2.19)	0.37 - 19.18	0.04 (0.98)	-5.67 - 5.18	0.49 (3.80)	-7.65 - 20.91
3	2.03 (1.58)	0.28 - 10.24	0.17 (0.85)	-2.91 - 4.31	0.11 (2.54)	-9.17 - 7.25
All	2.43 (2.52)	0.28 - 20.37	0.12 (1.23)	-5.90 - 7.35	0.12 (3.29)	-14.93 - 20.91
Cortical thickness						
1	0.69 (0.72)	0.13 - 3.88	-0.02 (0.91)	-4.61 - 2.23	-0.07 (0.98)	-4.89 - 2.53
2	0.71 (0.65)	0.10 - 3.40	-0.47 (0.97)	-5.42 - 1.44	-0.09 (1.20)	-6.81 - 2.96
3	0.59 (0.49)	0.11 - 2.68	-0.34 (1.67)	-7.09 - 3.34	-0.17 (0.53)	-2.26 - 0.95
All	0.66 (0.63)	0.10 - 3.88	-0.28 (1.24)	-7.09 - 3.34	-0.11 (0.94)	-6.81 - 2.96

[a] Measured with respect to average of 150 random values

[b] Measured with respect to corresponding non-random value
